# ddPCR increases detection of SARS-CoV-2 RNA in patients with low viral loads

**DOI:** 10.1007/s00705-021-05149-0

**Published:** 2021-07-12

**Authors:** Agnès Marchio, Christophe Batejat, Jessica Vanhomwegen, Maxence Feher, Quentin Grassin, Maxime Chazal, Olivia Raulin, Anne Farges-Berth, Florence Reibel, Vincent Estève, Anne Dejean, Nolwenn Jouvenet, Jean-Claude Manuguerra, Pascal Pineau

**Affiliations:** 1 Unité “Organisation nucléaire et Oncogenèse”, INSERM U993, Institut Pasteur, 28, rue du Docteur Roux, 75724 Paris, Cedex 15, France; 2 Cellule d’Intervention Biologique d’Urgence, Institut Pasteur, Paris, France; 3 Département de Virologie, Centre National de la Recherche Scientifique (CNRS) Unité Mixte de Recherche (UMR) 3569, Institut Pasteur, Paris, France; 4 Laboratoire de Biologie Médicale, Centre Hospitalier Compiègne-Noyon, Compiègne, France; 5 Laboratoire de Biologie Médicale, Groupe Hospitalier Nord-Essonne, Site de Longjumeau, Longjumeau, France; 6 Laboratoire de Biologie Médicale, Groupe Hospitalier Nord-Essonne, Site d’Orsay, Orsay, France

## Abstract

RT-qPCR detection of SARS-CoV-2 RNA still represents the method of reference to diagnose and monitor COVID-19. From the onset of the pandemic, however, doubts have been expressed concerning the sensitivity of this molecular diagnosis method. Droplet digital PCR (ddPCR) is a third-generation PCR technique that is particularly adapted to detecting low-abundance targets. We developed two-color ddPCR assays for the detection of four different regions of SARS-CoV-2 RNA, including non-structural (IP4-RdRP, helicase) and structural (E, N) protein-encoding sequences. We observed that N or E subgenomic RNAs are generally more abundant than IP4 and helicase RNA sequences in cells infected *in vitro*, suggesting that detection of the N gene, coding for the most abundant subgenomic RNA of SARS-CoV-2, increases the sensitivity of detection during the highly replicative phase of infection. We investigated 208 nasopharyngeal swabs sampled in March-April 2020 in different hospitals of Greater Paris. We found that 8.6% of informative samples (n = 16/185, *P* < 0.0001) initially scored as “non-positive” (undetermined or negative) by RT-qPCR were positive for SARS-CoV-2 RNA by ddPCR. Our work confirms that the use of ddPCR modestly, but significantly, increases the proportion of upper airway samples testing positive in the framework of first-line diagnosis of a French population.

**Supplementary Information:**

The online version contains supplementary material available at 10.1007/s00705-021-05149-0.

## Introduction

Seventeen years after the epidemics of severe acute respiratory syndrome (SARS), which originated in Foshan, in the southern Chinese province of Guangdong, another far more contagious coronaviral zoonosis emerged in December 2019 in the city of Wuhan, the capital of Hubei province (central China) [3, 9]. This novel disease, caused by a previously unknown member of the family *Coronaviridae*, was named SARS-CoV-2, after the initial SARS-CoV-1, with which it shares 82% nucleotide sequence identity [11, 22]. The disease itself was named coronavirus disease 2019 (COVID-19) by the World Health Organization on February 11, 2020.

Diagnosis of the disease is commonly based on amplification by reverse transcription quantitative PCR (RT-qPCR) of at least two different fragments of the SARS-CoV-2 RNA genome [13]. The clinical sample type and the time since symptom onset are critical parameters for COVID-19 diagnosis [34]. SARS-CoV-2 replication is characterized by an apparent downward migration from epithelia in the nasal cavity to those of the throat, and then to pulmonary alveoli. Viral genome copy numbers therefore tend to be higher in the nasopharyngeal compartment at the onset of symptoms before declining progressively [19, 35]. In severe forms of COVID-19, in which patients display SARS-CoV-2 RNA in different organs or fluids, the viral genome is more readily detected in lower respiratory tract samples such as bronchoalveolar lavage fluid (BALF) or sputum than in extracts from upper respiratory tract locations [35]. The infection, however, can extend well beyond the respiratory tract and, although SARS-Cov-2 genomic RNA is infrequently detected in patient blood samples, evidence for protracted viral shedding in feces is found by rectal swab analysis in a significant proportion of patients [36, 38].

Nevertheless, COVID-19 diagnosis is unfortunately characterized by a significant proportion of false negative results, estimated to be 20-40% [20, 21]. This situation has been emphasized from the onset of the pandemic by radiologists using thoracic CT scan, which is claimed to be more sensitive than quantitative real-time PCR detection. Different factors are presumably responsible for these failures. Early sampling after symptom onset improves detection rates, but the level of viral replication in the upper respiratory tract is often relatively low at the time of patient presentation [27]. Second, for the same patient at a given sampling site, viral replication levels fluctuate, as seen in most initial descriptions of COVID-19. Consequently, in a significant proportion of cases, the sequence of sampling outcomes for a given patient is marked by a succession of positive and negative results [5, 39].

The improvement of virus detection in COVID-19 cases thus remains a priority in clinical practice, both to diagnose new cases and to authorize discharge of the patient. Sensitive detection further aids in better tracking of virus circulation between index cases and their contacts, identifying asymptomatic, presymptomatic or paucisymptomatic patients, and detecting the infectious agent in samples such as blood and urine, which are deemed to have low levels of SARS-CoV-2 RNA. Several technical options, including loop-mediated isothermal amplification (LAMP), which can be coupled with CRISPR-Cas technology, have been developed recently and represent additional potentially promising advancements in COVID-19 diagnosis [2, 4].

Droplet digital PCR (ddPCR) is a third-generation PCR technique based on an initial step of sample partitioning that produces a stable emulsion of 15,000-20,000 droplets of around 1 nL each. Subsequently, 40 cycles of classical PCR are carried out in each of these impervious nanocompartments, which are then analyzed individually using a specific fluorescence reader for the presence of an amplification signal. ddPCR has been used for COVID-19 diagnosis and sometimes even as a reference technique to compare various RT-qPCR kits [12, 28]. However, it has rarely been used retrospectively on a series of RT-qPCR-negative samples to provide an estimate of the proportion of false negative results [40]. Here, we have leveraged our experience with ddPCR for detecting low-abundance targets (from somatic tumor mutations, or viruses from patients with liver cancer [24, 25]) for the analysis of SARS-CoV-2 samples. We present an assessment of ddPCR on different SARS-CoV-2 targets (envelope, nucleocapsid, RNA-dependent RNA polymerase, RdRP, and helicase genes) and its application to a group of RT-qPCR-negative patients from selected Greater Paris hospitals.

## Materials and methods

### Patients

Nasopharyngeal samples (n = 208) from suspected cases of COVID-19 were obtained within the framework of our mission as the “Cellule d’Intervention Biologique d’Urgence”, a surveillance laboratory created by the Ministry of Health for biological emergency response and located at Institut Pasteur. This laboratory receives human samples for diagnostic and surveillance purposes as well as for the development of new detection techniques. For such use, no ethical approval is required under French Law. Samples were obtained from routine medical investigations, and all patients were primarily processed using state-of-the-art diagnostic procedures established for COVID-19. In addition, necropsy samples from three patients who died from COVID-19-associated encephalopathy were analyzed. Different brain regions were sampled in each case: cranial nerves (olfactory bulb and tract, trigeminal nucleus), brain stem (*medulla oblongata,* periaqueductal gray), diencephalon (hypothalamus), cerebrum (*splenium corpus callosum*, middle frontal, and middle temporal gyri).

## RNA extraction

Following sampling, nasopharyngeal swabs from suspected cases of COVID-19 were stored in sterile vials and soaked in 1.5 mL of viral transport medium (VTM). VTM (pH = 7.3) includes Hank’s balanced salt solution, bovine serum albumin, gelatin, sucrose, cystine, glutamic acid, amphotericin B, vancomycin, colistin, and phenol red. A volume of 100 to 400 μL of VTM was extracted using either Tri-Reagent LS (MRC, Cincinnati, OH, USA) or a NucleoSpin Dx Virus viral nucleic acid isolation kit (Macherey-Nagel, Düren, Germany) according to the manufacturer's instructions (see Supplemental Data for details).

## Quantitative real-time PCR

All patients were tested by RT-qPCR in the course of routine medical diagnosis for COVID-19. Sample RNA extracts were analyzed using a SARS-CoV-2 real-time gene duplex RT-qPCR (IP2-nsp9, and IP4-RdRP) developed by the French National Reference Centre for Respiratory Viruses and a real-time E gene RT-PCR from the Charité protocol (see WHO Coronavirus disease COVID-19 technical guidance: Laboratory testing for 2019-nCoV in humans, available at https://www.who.int/docs/default-source/coronaviruse/whoinhouseassays.pdf) [6, 19]. Samples were considered SARS-CoV-2 positive if at least two out of three SARS-CoV-2 gene targets were detected by the RT-qPCR assays. Out of the 252 samples investigated, 228 (90.4%) were considered negative or inconclusive (n = 8, 3.1%) by qPCR, and 16 (6.3%) were positive for SARS-CoV-2 RNA by qPCR.

## Droplet digital PCR

Several reverse transcription kits were compared and used according to the manufacturer’s instructions. The comparison included SuperScript III (Invitrogen), and Superscript IV (Invitrogen), High Capacity Reverse Transcription Kit (Applied Biosystems, Foster City, CA, USA), Prime Script (Takara Bio, Kusatsu, Japan), iScript (Bio-Rad, Hercules, CA, USA), and iScript Advanced cDNA Synthesis Kit (Bio-Rad). RNA volumes ranging between 5 and 14.8 μL were used for reverse transcription. With regard to the two-step procedure that uses independent reverse transcription and subsequent ddPCR, among the six RT kits used, the iScript™ Advanced cDNA Synthesis Kit (Bio-Rad) consistently produced higher numbers of positive droplets when tested on dilutions (in distilled water or in human cell line RNA solution corresponding to the median concentration obtained in swab samples, *i.e.*, 2.5 ng/μL) of a sample positive for SARS-CoV-2. The RT step was adapted to each target through the addition of random, oligo-dT, and virus-specific primers in different combinations and/or concentrations in order to increase ddPCR yields (cf Supplementary Table S1 and Supplementary Fig. S2). The assay targeting the RdRP (IP4) gene used the same primers and probe as used for RT-qPCR in the initial diagnostic procedure.

Finally, to maintain relevance to procedures that are generally used in clinical laboratories, we employed a one-step RT-ddPCR kit (Bio-Rad) corresponding to a single-step version of iScript™ Advanced. One of the major advantages of this procedure is that it allows the direct addition of 10 μL of extracted RNA in the ddPCR, thus increasing the probability of detecting the virus when compared to the 2-step procedure, which allows the addition of a maximum of 2.5 μL of RNA.

**Fig. 1:**
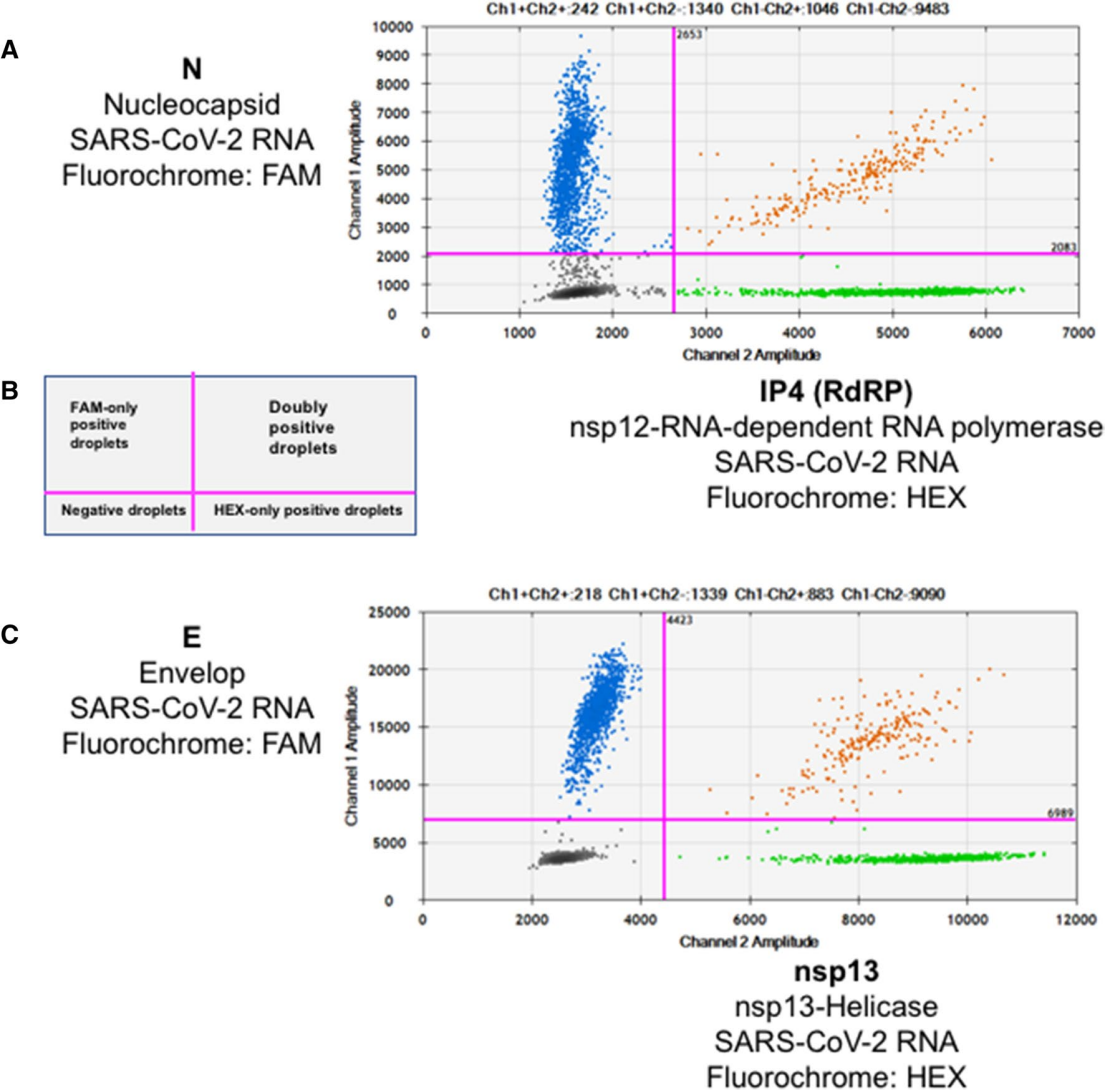
Representative examples of single-step (RT + ddPCR) amplification of SARS-CoV-2 RNA. (A) Dual-color ddPCR detection of nucleocapsid (N) and IP4 (RdRP) sequences of SARS-CoV-2 RNA in a clinical sample (n#200132). Each dot on the figure represents a single droplet. The *x*- and *y*-axes represent the HEX and FAM fluorescence levels, respectively, for each droplet present in the space of analysis. Ultra pink lines defining quadrants represent the background thresholds of each fluorescent dye. (B) Schematic view of a ddPCR result with its interpretation in each quadrant. (C) Dual-color ddPCR detection of envelope (E) and nsp13 sequences of SARS-CoV-2 RNA in the same clinical sample used in panel A.

The primers and probes that were used, many of which have been published elsewhere [6, 19, 33], are listed in Supplementary Table S1. We developed ddPCR assays for four viral targets: the envelope (E), nucleocapsid (N), RNA-dependent RNA polymerase (RdRP, nsp12), and helicase (nsp13) genes. The optimal primer and probe concentrations were determined for each assay. All probes that were labeled with FAM or with HEX fluorescent dyes were modified at the 3’ end by addition of a dark quencher, Iowa Black quencher (IBQ, Integrated DNA Technologies, Coralville, Iowa, USA), which does not emit light and generates low background (Supplementary Table S1). Droplet digital PCR reactions were performed on a QX200 system (Bio-Rad) using a One-Step RT-ddPCR Advanced Kit according to the manufacturer’s instructions (Bio-Rad). The optimal combinations of targets in the single-step procedure were N + RdRP (IP4, nsp12) and E + helicase (nsp13), as shown in Figure 1. In brief, 10 μL of RNA was added to 5 μL of ddPCR Supermix, 1 μL of 300 nM DTT, and 2 μL of reverse transcriptase in a final volume of 20 μL. RT and PCR amplification were carried out in an ICycler PCR instrument (Bio-Rad) with the following steps: 1 cycle of 25°C for 3 min, 50°C for 60 min, and 95°C for 10 min, followed by 40 cycles of 95° for 30s and 55°C (N/IP4) or 59°C (E/nsp13) for 1 min, and a termination step of 98°C for 10 min. All cycles were performed with a ramping rate of 2°C/s.

The limit of detection (LoD) determination was based on the establishment of the limit of the blank (LoB), which is defined as the maximal number of positive droplets obtained on pre-pandemic RNA samples negative for SARS-CoV-2, calculated according to a modified version of the procedure described by Armbruster and Pry [1, 23]. The four LoB values determined for amplification of the RdRP-IP4 (n = 3), helicase-nsp13 (n = 3), E (n = 3), and N (n = 4) genes were derived from the analysis of 110 ddPCR replicates from 65 different SARS-CoV-2-negative RNA samples extracted from human nasopharyngeal specimens collected during the pre-COVID-19 era that generated a mean droplet number of 16,188 ± 1317. The LoD was established for each SARS-CoV-2 target gene analyzed, following the same guidelines, and was set at five droplets for all tests.

## Selection of reference genes

To control RNA quality, clinical samples were tested for four different cellular genes: glyceraldehyde phosphate dehydrogenase (*GAPDH)*, hypoxanthine phosphoribosyltransferase 1 (*HPRT1),* receptor for activated C kinase 1 (*RACK1*), and ribosomal protein p30 (*RPP30*). *RACK1*, the most strongly expressed of the tested set, was used as the preferred reference gene (see Supplementary Fig. S3) [26, 29].

## Virus amplification and flow cytometry

The strain BetaCoV/France/IDF0372/2020 was supplied by Prof. S. van der Werf, Head of the National Reference Centre for Respiratory Viruses of Institut Pasteur (Paris, France). Viral stocks were titrated on Vero E6 cells using plaque assays.

Human A549-ACE2 cells, which have been modified to stably express ACE2 via lentiviral transduction, were obtained from Prof. Olivier Schwartz, (Institut Pasteur, Paris, France). Calu-3 (HTB-55, human lung adenocarcinoma cells), CaCo-2 (HTB-37, human colorectal adenocarcinoma), and Vero E6 cells were purchased from ATCC and maintained at 37°C in a humidified atmosphere with 5% CO_2_. Calu3 and Vero E6 cells were cultured in DMEM (Sigma) supplemented with 10% FBS (Gibco) and 100 U of penicillin-streptomycin (Thermo Fisher Scientific) per ml. The medium for Calu3 cells was further supplemented with 1 mM sodium pyruvate (Thermo Fisher Scientific) and 10 mM HEPES (Thermo Fisher Scientific).

Cells were fixed in 4% paraformaldehyde (PFA) for 15 to 30 minutes at room temperature, and staining was performed using a solution containing 1% BSA, 0.05% sodium azide, and 0.05% saponin in PBS. Cells were labeled with primary antibodies against the coronavirus spike protein (SARS_Ssd3 293, a kind gift from Dr. Nicolas Escriou, Institut Pasteur, Paris, France), and then with secondary antibodies (Alexa Fluor 647–labeled anti-mouse), for 30 minutes at room temperature. Surface staining was performed before fixation, in PBS with 1% BSA. Cells were incubated with primary antibodies, and then with secondary antibodies, for 30 minutes at room temperature. Cells were fixed for 15 minutes in 4% PFA and analyzed on an Attune NxT Flow Cytometer (Thermo Fisher) with FlowJo software.

## Data analysis and statistical tests

ddPCR data were analyzed using QuantaSoft™ software (version 1.7.4, Bio-Rad). Statistical analysis was performed using the Prism 8.4.2 statistical package (GraphPad, San Diego, CA, USA). Numerical variables are reported as the mean and standard deviation or median and interquartile range, according to their distribution type (normal or not). Data were compared using Student’s *t*-test or the Mann-Whitney U test, as appropriate. Categorical variables were summarized as frequencies and compared using Fisher’s exact test. All tests were two-sided, and the level of significance was set at *P* < 0.05.

## Results

### Development of an in-house ddPCR assay

We compared our in-house ddPCR test with the Bio-Rad kit (catalog no. 1200802), which targets two different fragments of the SARS-CoV-2 N gene (N1 and N2), with *RPP30* mRNA as a control. Overall, our results obtained using 15 SARS-CoV-2-positive samples were consistent with those obtained using the Bio-Rad kit. We noticed, however, that in the higher or lower ranges of SARS-CoV-2 RNA concentrations, interpretation of commercial kit results can be more complex due to the constitutive presence of *RPP30*-positive droplets representing a third type of amplicon that “geographically” interferes with virus-positive droplets spread in a two-dimensional space (see Supplementary Fig. S4).

We next compared ddPCR and RT-qPCR performed using the same primer/probe combinations on a dilution series of two different samples and found ddPCR to be more sensitive and reliable than RT-qPCR at very low target numbers (Table 1).

**Table 1:** Comparisons of RT-qPCR and ddPCR results obtained using serial dilutions of SARS-CoV-2-infected samples and negative controls

Samples	RT-qPCR (Cq)		ddPCR (droplets)		
	N	RdRP (IP4)	N positive	RdRP (IP4) positive	Accepted
A549 24H 1.0 E-02	15.99	16.01	13566	13566	13566
A549 24H 1.0 E-03	19.09	19.23	15106	15106	15106
A549 24H 1.0 E-04	22.24	22.53	12839	12839	12839
A549 24H 1.0 E-05	25.64	26.01	8435	3626	13203
A549 24H 1.0 E-06	29.20	29.26	1114	350	11081
A549 24H 1.0 E-07	32.60	32.90	160	45	13507
A549 24H 1.0 E-08	34.94	37.39	15	2	12290
200132 E-03	23.36	21.79	6415	6332	10286
200132 E-04	27.14	25.62	5075	4256	13375
200132 E-05	29.65	28.11	783	564	12615
200132 E-06	32.90	31.44	34	32	11851
200132 E-07	38.07	37.33	45	34	14180
200132 E-08	N/A	N/A	1	1	15403
293T (1)	39.54	N/A	0	0	12993
293T (2)	N/A	N/A	1	0	11477
H_2_0	N/A	N/A	0	0	13076

Tenfold dilutions of RNA extracts from an infected cell line (A549-ACE2) and a patient (n#200132) diagnosed as strongly positive for SARS-CoV-2 RNA were compared using RT-qPCR and ddPCR. Accepted droplets correspond to the total count of *bona fide* droplets as detected in a given well by the droplet reader. RNA extracted from uninfected 293T cells was used as a negative control.

## Sensitivity of ddPCR for different regions of the SARS-CoV-2 genome

In addition to the full-length genome, replication of coronaviruses produces a set of subgenomic RNA (sgRNA) molecules with heterogeneous 5’ ends but identical 3’ ends. The nine major sgRNAs of SARS-CoV-2 are encoded by the last third of the viral genome, whereas ORF1a-b sequences, encoding non-structural proteins (nsps), are present exclusively in full-length genomic viral RNAs. All genomic and subgenomic SARS-CoV-2 RNAs therefore contain the nucleocapsid (N) gene sequence due to its position at the 3’ end of the viral genome. For this reason, N likely represents the most abundant and the most suitable target for SARS-CoV-2 RNA detection [17], although it is far from being used systematically in clinical practice.

**Fig. 2:**
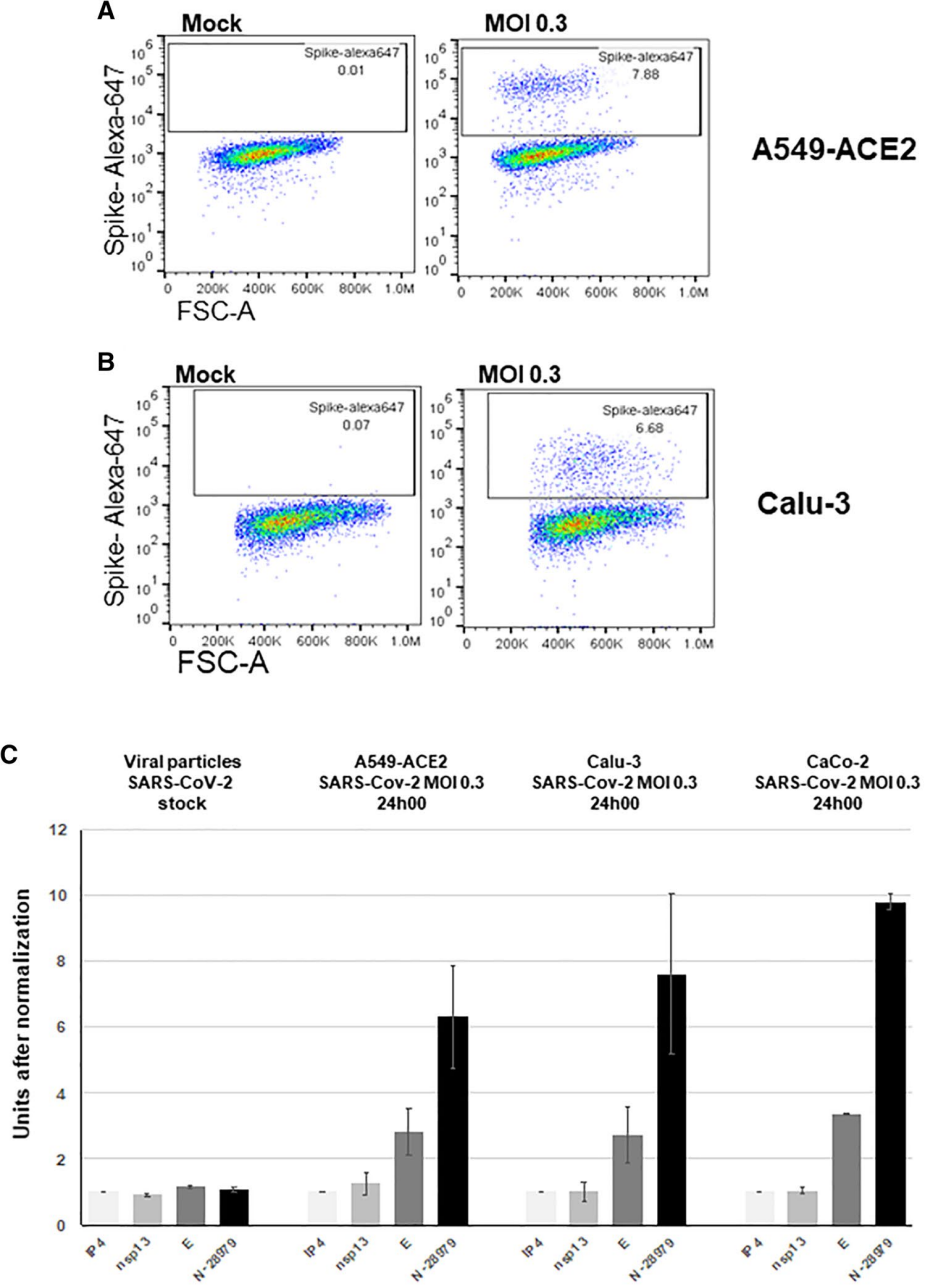
(A) Flow cytometric analysis of A549-ACE2 and (B) Calu-3 cells infected with SARS-CoV-2. The cells were infected at an MOI of 0.3 for 24 hours. Surface S staining of infected cells was analyzed by flow cytometry. The results are representative of two independent experiments. The percentage of cells positive for the viral spike protein is indicated in the top right corner. (C) Normalized detection rates by ddPCR of different targets on SARS-CoV-2. The number of positive droplets obtained by RdRP-IP4 amplification was used as a reference. Viral particle stocks were obtained from Vero cells.

A549-ACE2 and Calu-3 cells were infected with SARS-CoV-2 stocks obtained from Vero cells. Virus replication was assessed by analyzing the intracellular presence of the viral spike protein by flow cytometry at 24 h after infection at an MOI of 0.3. About 8.0% of A549-ACE2 and 7.0% of Calu-3 cells were infected (Fig. 2A and B).

We then compared the ddPCR results obtained for different regions of the viral genome to assess whether this technique is capable of distinguishing mRNA from active replication from that of genome-loaded viral particles. For this, two targets mapping to the 5’ non-structural part of the genome (RdRP-IP4-nsp12 and helicase-nsp13) and two others located in structural-protein-encoding genes (envelope [E] and nucleocapsid [N]) in the 3’ part of SARS-CoV-2 genome were chosen and tested on four types of RNA samples: RNA extracted from SARS-CoV-2 stocks obtained from a Vero cell culture, and RNAs extracted from A549-ACE2, Calu-3, and CaCo-2 cells exposed to SARS-CoV-2 for 24 h at an MOI of 0.3. As expected, and unlike the virus particle stocks, we consistently found structural genes, in particular N, to be detected in larger quantities than nsps-encoding genes in infected cells (Fig. 2C). These findings, presumably the result of the larger amount of sgRNAs present in replicating host cells, thus prompted us to select the N gene as the principal target for SARS-CoV-2 detection by ddPCR in clinical samples. N gene detection was associated in a dual-color assay with RdRP-IP4 detection, but it was, in our hands, incompatible with concomitant amplification of E gene. By contrast, E gene amplification was compatible with helicase gene (nsp13) amplification.

## Testing of patients for SARS-CoV-2 RNA

A series of 208 samples from the respiratory tract (95% as nasopharyngeal swabs) arrived in the framework of the on-call activity of the Cellule d’Intervention Biologique d’Urgence (CIBU) between the March 14 and April 13, 2020. Patients were recruited from hospitals in a large area around Paris (Compiègne, Longjumeau, and Orsay). A subset of 13 samples (6.2%) had been scored as clearly positive for SARS-CoV-2 by RT-qPCR, while the remaining 195 were either undetermined (n = 8, 3.8%) or negative (n = 187, 89.9%). The clinical and biological features of the patients are summarized in Supplementary Table S2. A subset of 16.9% (n = 35/208) of patients tested did not exhibit any respiratory symptoms (*e.g*., dyspnea, flu-like syndrome, fever, coughing, ARD, or anosmia-ageusia) evocative of COVID-19. Likewise, in a large subset of subjects tested (31%, 64/207), prior contact with COVID-19 patients was documented.

A further series of 44 RNAs extracted from different brain regions of three patients who died from COVID-19-associated encephalitis was similarly analyzed for the presence of SARS-CoV-2 RNA.

## Sample quality assessment

We first decided to assess the quality of the RNA extracted from clinical specimens, as the quality of the sample is known to be crucial for obtaining a consistent result. However, SARS-CoV-2 is known to strongly suppress host-cell transcription and therefore it is necessary to identify expressed cellular genes as controls for checking RNA quality of coronavirus-infected cells [17]. We thus tested respiratory samples for the presence of *RACK1* (receptor for activated C kinase 1) mRNA, which is more highly and stably expressed in airway cells than other well-known candidates (*GAPDH, HPRT1, RPP30*). Its absence thus represents a conservative indicator for the unsuitability of the biological material. A small subset of RNAs from airway samples (n = 10/208, 4.8%), all initially negative for SARS-CoV-2 RNA, was also negative for *RACK1* mRNA. This situation implies that, in the case of a negative result for SARS-CoV-2 amplification using these samples, we may conclude neither the presence nor the absence of viral RNA. These samples were excluded from further analysis. A similar approach was used for brain tissue, using *HPRT1* (hypoxanthine phosphoribosyl transferase 1) mRNA as a target. Only two brain samples were negative (n = 2/44, 4.5%) for *HPRT1* mRNA.

## Sensitivity of SARS-CoV-2 RNA detection in clinical samples by ddPCR

**Fig. 3:**
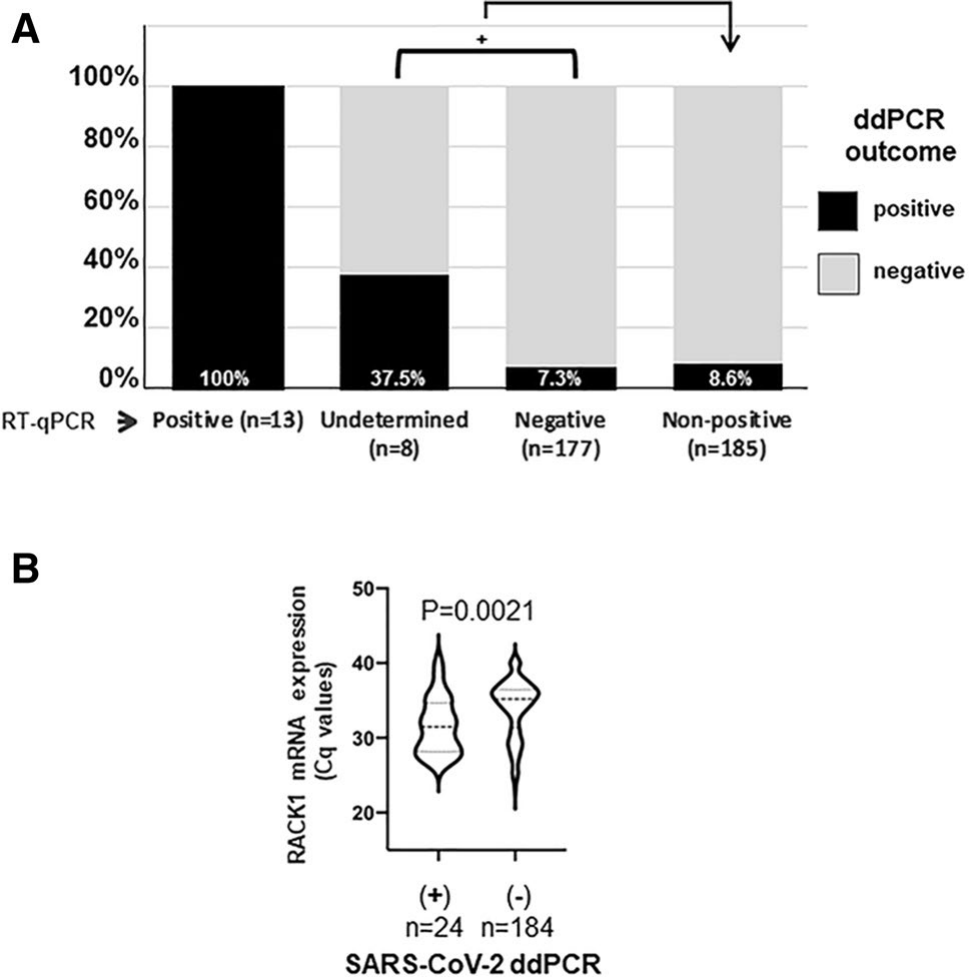
(A) ddPCR results for three sample categories based on initial qRT-PCR test results. (B) Comparison of quantification cycles (Cq) for RACK1 mRNA in airway RNA samples from ddPCR-positive and ddPCR-negative samples

For RNA extracted from respiratory tract specimens, 13 out of 13 (100.0%) samples that were initially SARS-CoV-2 RNA positive by RT-qPCR were confirmed, two out of eight (25.0%) undetermined samples were shown to be positive, and another remained undetermined due to a low positive droplet number for the N gene (n = 1/8, 12.5%). Within the initially negative subset, eight out of the 177 informative samples (4.5%) were found to contain SARS-CoV-2 RNA levels above the LoD (≥ 6 droplets/reaction) on both viral targets (N and RdRP-IP4) (Fig. 3 and Table 2). Five additional samples (n = 5/177, 2.8%) yielded droplets above the LoD for a single viral target (N in four cases and IP4 in the remaining one). Overall, 8.6% (16/185) of the patients initially scored as “non-positive” by RT-qPCR yielded a positive signal with ddPCR, i.e., a very significant difference (*P* = 2.17 E-05) from the initial diagnosis. Moreover, we found that droplets that were positive for the N gene were more numerous than those positive for RdRP (IP4) in nine out of 16 cases (56.2%).

**Table 2:** Clinical and biological features of initially non-positive patients who became positive upon ddPCR analysis

ID	Age	Sex	Distance from symptoms onset (in days)	RT-gPCR	RT-gPCR SARS-CoV-2			ddPCR SAR S-CoV-2										Antecedents		
				^*Cq*^* RACK1*	^RT-qPCR^ diagnosis	Cq E	^cq RdRP (IP4)^	N(+) droplets	RdRP(+) (IP4) deoplets	Accepted droplets	Lymphopenia (pos=1, neg=0)	CRP (mg/L)	Respiratory	ARDS	Fever	Cough	Cardiovascular	Diatebetes	Tumor disease	Disease outcome
200307	59	M	14	37.2	neg	>37	>37	10	5	22926	0	231	1	0	1	0	1	1	0	discharged
200325	51	F	2	35.3	neg	>37	>37	57	79	17929	0	<6	1	0	1	0	0	0	0	discharged
200327	15	F	3	33.0	neg	>37	>37	37	8	16217	1	<6	1	1	1	0	1	0	0	discharged
200352	54	M	8	33.1	neg	>37	>37	62	83	16762	1	100	1	0	1	0	0	1	0	discharged
200397	83	M	na	35.4	neg	>37	>37	16	7	17599	1	97	0	0	0	0	1	1	1	discharged
200400	62	F	na	35.5	undetermined	>37	33.9	2	7	11361	1	12	0	0	1	1	1	0	1	discharged
200401	85	F	7	36.5	neg	>37	>37	13	5	19345	1	86	1	0	0	0	1	1	0	discharged
200549	55	M	11	36.5	neg	>37	>37	6	0	21163	na	na	1	0	0	1	1	0	0	discharged
200594	50	F	1	31.3	undetermined	35.2	31.9	177	70	10500	na	na	0	0	1	0	0	0	0	discharged
200598	73	M	2	27.9	neg	>37	>37	583	669	9061	0	<6	0	0	1	0	1	0	0	discharged
200600	40	F	5	28.8	undetermined	35.8	32.9	120	163	12474	0	<6	1	0	1	0	0	0	0	discharged
200602	74	M	8	30.0	neg	>37	>37	6	9	3267	1	220	1	0	1	1	1	1	0	discharged
200677	22	M	2	29.9	neg	>37	>37	0	7	20918	1	64	1	0	1	1	0	0	0	discharged
200678	66	M	1	26.6	neg	>37	>37	8	6	19563	0	142	1	0	1	0	1	0	1	discharged
200692	72	M	22	27.4	neg	>37	>37	126	81	8770	1	27	0	1	0	0	1	1	0	deceased
200728	75	M	6	32.5	neg	>37	>37	9	3	12688	1	123	1	1	0	0	0	0	0	**deceased**

The presence or absence of symptoms is indicated by 1 or 0, respectively.

As to sample quality, we noted that mean values for quantification cycles (Cq) of *RACK1* were significantly lower in respiratory samples that were positive for SARS-CoV-2 RNA (Cq, mean ± SD = 31.5 ± 3.7) when compared to negative samples (34.0 ± 3.8, *P* = 0.0024, Fig. 3B), indicating, as stressed by others, that sample quality is a decisive parameter for virus detection [18].

For SARS-CoV-2 detection in brain tissues, three samples from a single patient were found positive by ddPCR. Positive tissues included the olfactory bulb, olfactory tract, and the middle frontal gyrus, whereas all other tissues tested negative.

## Clinical and biological features of patients with SARS-CoV-2

We next asked whether patients who were positive for SARS-CoV-2 RNA by ddPCR differed from those who remained negative. Out of the 16 patients with positive ddPCR, fourteen recovered (87.5%), but two died (12.5%) after 5 and 8 days of hospitalization, respectively (Table 2). We also found that a substantial proportion (64.2%, n = 9/14) of the patients who were positive by ddPCR exhibited some degree of lymphopenia (<1000 lymphocytes/μL), while 71.4% (n = 10/14) displayed increased plasma levels of C-reactive protein (>6mg/L). CT imaging analysis indicated that 60.0% (n = 6/10) of the patients presented with a lung parenchyma pattern evocative of COVID-19. This was also the case for the two deceased patients (40% and 75% of lung tissue affected).

**Fig. 4:**
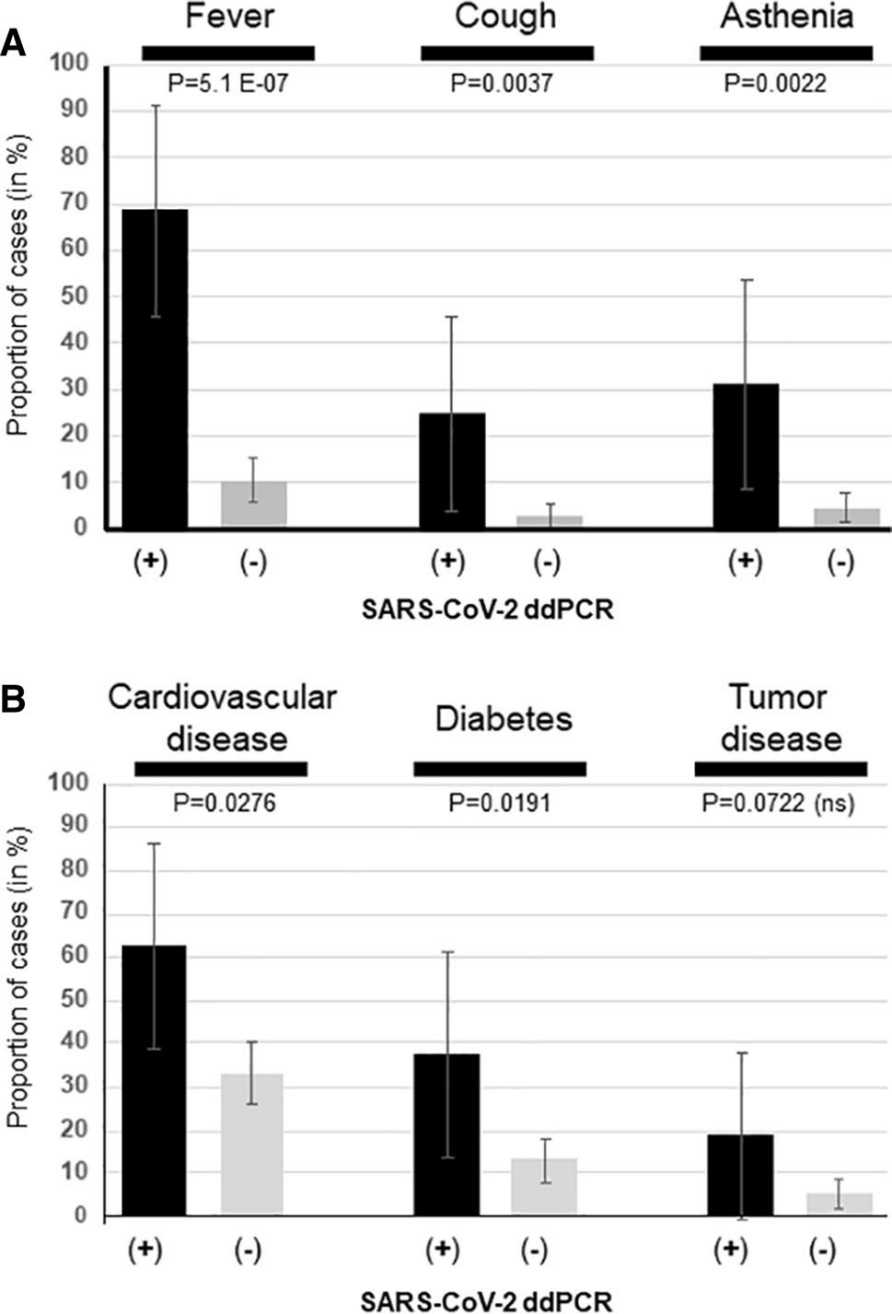
(A) Prevalence of a few symptoms according to ddPCR outcome. (B) Prevalence of co-morbidities according to ddPCR outcome

We next compared the 16 patients who were positive for SARS-CoV-2 RNA with the 169 who remained completely negative despite the apparent suitability of their samples for viral diagnostic testing as revealed by the presence of RACK1 mRNA. Indeed, the prevalence of several symptoms was statistically different between these 16 patients and the others. This was the case for fever (68.7% vs. 10.6%, OR = 18.1, 95% CI = 5.8-50.9, *P* = 5.1 E-07), cough (25.0% vs. 2.9%, OR = 10.9, 95%CI = 2.9-44.3, *P* = 0.003), and asthenia (31.2% vs. 4.7%, OR = 9.1, 95%CI = 2.7-32.5, *P* = 0.002) (Fig. 4A). Regarding medical antecedents, cardiovascular disease was found in 62.5% of positive patients vs. 33.1% of negative patients (OR = 3.3, 95% CI = 1.2-9.7, *P* = 0.027). The prevalence of diabetes (37.5% vs. 13.0%, OR = 4.0, 95% CI = 1.2-11.7, *P* = 0.019) and tumor disease (18.7% vs. 5.3%, OR = 4.1, 95% CI = 1.07-15.7, *P* = 0.072, ns) were also elevated in positive patients, albeit non-significantly for the latter group (Fig. 4B). Overall, patients who were negative by RT-qPCR but positive by ddPCR for SARS-CoV-2 RNA appeared more often to be affected by a severe form of COVID-19 and/or to exhibit symptoms and selected co-morbidities than SARS-CoV-2 RNA-negative subjects.

## Discussion

The issue of SARS-CoV-2 RNA detection in biological samples is of considerable importance and turned out to be problematic from the onset of the pandemic. In medical practice, it is required for management, monitoring, and medical procedures applied to patients with severe respiratory disease. It is also necessary for their safe discharge in the resolution phase. Outside of medical institutions, when the disease is benign, a positive test for SARS-CoV-2 RNA implies that the patient must self-isolate to avoid further spread of the virus. In public health, SARS-CoV-2 RNA detection is important for identifying transmission clusters, for monitoring virus circulation by calculating a realistic reproduction number (R), and for estimating the burden of COVID-19-associated morbidity and mortality. Serological tests are often considered an adequate surrogate for molecular detection. However, it appears that the sensitivity of antibody detection is still too low in the initial phase of infection for it to be used to diagnose an acute SARS-CoV-2 infection [8]. Likewise, the sensitivity of computed tomography (CT), once claimed to surpass that of PCR, turned out to be suboptimal and time-consuming when used alone [16].

As a consequence, the problem of false-negative COVID-19 diagnosis has received considerable attention since the onset of the pandemic. The true proportion of false-negative PCR results is difficult to estimate. This is primarily due to the fact that the causes of false-negative qPCR results are diverse. In most cases, a false-negative result is either due to absence of the virus in the region sampled or to inadequate sampling rather than failure of RT-qPCR to detect viral RNA [18]. In the case of low-abundance targets, ddPCR is, of course, advantageous for increasing diagnostic sensitivity. However, pre-analytical factors such as the volume of initial sample used for RNA extraction or the amount the clinical sample is diluted before testing represent additional critical parameters that must be taken into consideration [10, 12, 33]. Similarly, when viral loads are low, limit of blank and limit of detection reductions are also critical for sensitive detection of SARS-CoV-2 RNA. These parameters are specific for each combination of primers and probes. For this reason, we here modified fluorescent probes with a dark quencher (Iowa Black) to reduce fluorescent background in both two-step and single-step reactions.

Overall, with the present series of samples, we provide a real-world assessment of the capacity ddPCR to detect false-negative samples. Our ddPCR assays confirmed 100% of the samples that initially gave positive results by qPCR and changed, with high confidence, the status of around 8-9% of samples previously considered non-positive for SARS-CoV-2 RNA. This latter ratio might be viewed as rather low but needs to be placed in the context of the initial phase of the pandemic, in which 17% and 31% of patients tested displayed neither overt symptoms nor evidence of previous contact with COVID-19 patients, respectively. A more targeted series might have yielded substantially higher rates of positive PCR results, so we consider 8-9% to represent a lower boundary for false-negative results. Our work is in agreement with that of Yu and coworkers, who, using ddPCR, observed a rate of 0.9% false-negative nasal and throat swabs (n = 1/112) and 32.0% of positive samples among undetermined samples (n = 8/25) [40]. In light of our results, we would suggest the use of ddPCR for samples considered “undetermined” by qPCR or for negative nasopharyngeal samples obtained long after symptom onset (>10 days) in the context of high COVID-19 probability substantiated by evocative symptoms, positive chest imagery, or previous contact with confirmed cases.

In addition, we provide data concerning the quantification of amplification products from structural and non-structural SARS-CoV-2 genes with the aim of estimating the replicative activity of the virus. These results should be confirmed on a larger series and positioned in a more detailed clinical landscape in order to properly assess the role of the ratio of structural to non-structural genes as a biomarker of replicative infection. In the current series of clinical samples, however, ddPCR targeting a structural-gene-encoding RNA might be slightly more sensitive than targeting a non-structural gene (more droplets for N than for RdRP in 56% of positive cases).

In conclusion, our work showed, in a French context, that ddPCR might increase by 8.6% the number of positive COVID-19 cases diagnosed. This could be extrapolated to 5.5 million additional cases worldwide using the incidence data from December 3, 2020. Together with the use of a third-generation PCR technique, improvements in the molecular detection of SARS-CoV-2 can be achieved at different pre-analytical and analytical steps of the process, including proper sampling, an increased concentration factor between virus stabilization medium and extracted RNA resuspension, the choice of reverse transcription and qPCR amplification kits, probe characteristics, and the choice of the viral gene target in the SARS-CoV-2 RNA. However, due to the limited availability of ddPCR instruments and the higher complexity of the method when compared to classical RT-qPCR, it remains questionable whether this technique is applicable for routine high-throughput medical practice. ddPCR should be preferably targeted to severe COVID-19 cases with fluctuating viral loads, to remove doubt about undetermined cases with a single positive viral target, or before hospital discharge of convalescent hospitalized patients to ascertain their negativity [7, 30, 37]. Other medical applications to be considered include ascertainment of donor negativity before transplantation or, for example, ensuring the negative status of the crew before boarding a marine vessel [14, 32]. ddPCR could further be implemented for the environmental surveillance of wastewater or for indoor air monitoring [15, 31]. Finally, we highlight that inclusion of the SARS-CoV-2 nucleocapsid (N) sequence, a marker of viral replication, as a primary target in first line diagnosis will increase the sensitivity of PCR tests.

## Supplementary Information

Below is the link to the electronic supplementary material.

Supplementary file1 (PDF 477 KB)

Supplementary file2 (DOCX 198 KB)

## Data Availability

Data are available from the authors on request.
