# Supplementary material for: ddPCR increases detection of SARS-CoV-2 RNA in patients with low viral loads
Source: Arch Virol. 2021 Jul 12;166(9):2529–40. doi: 10.1007/s00705-021-05149-0 (PMC8273560; doi:10.1007/s00705-021-05149-0)
Supplement: Supplementary file 1 — Supplementary file1 (PDF 477 KB) [file 705_2021_5149_MOESM1_ESM.pdf]

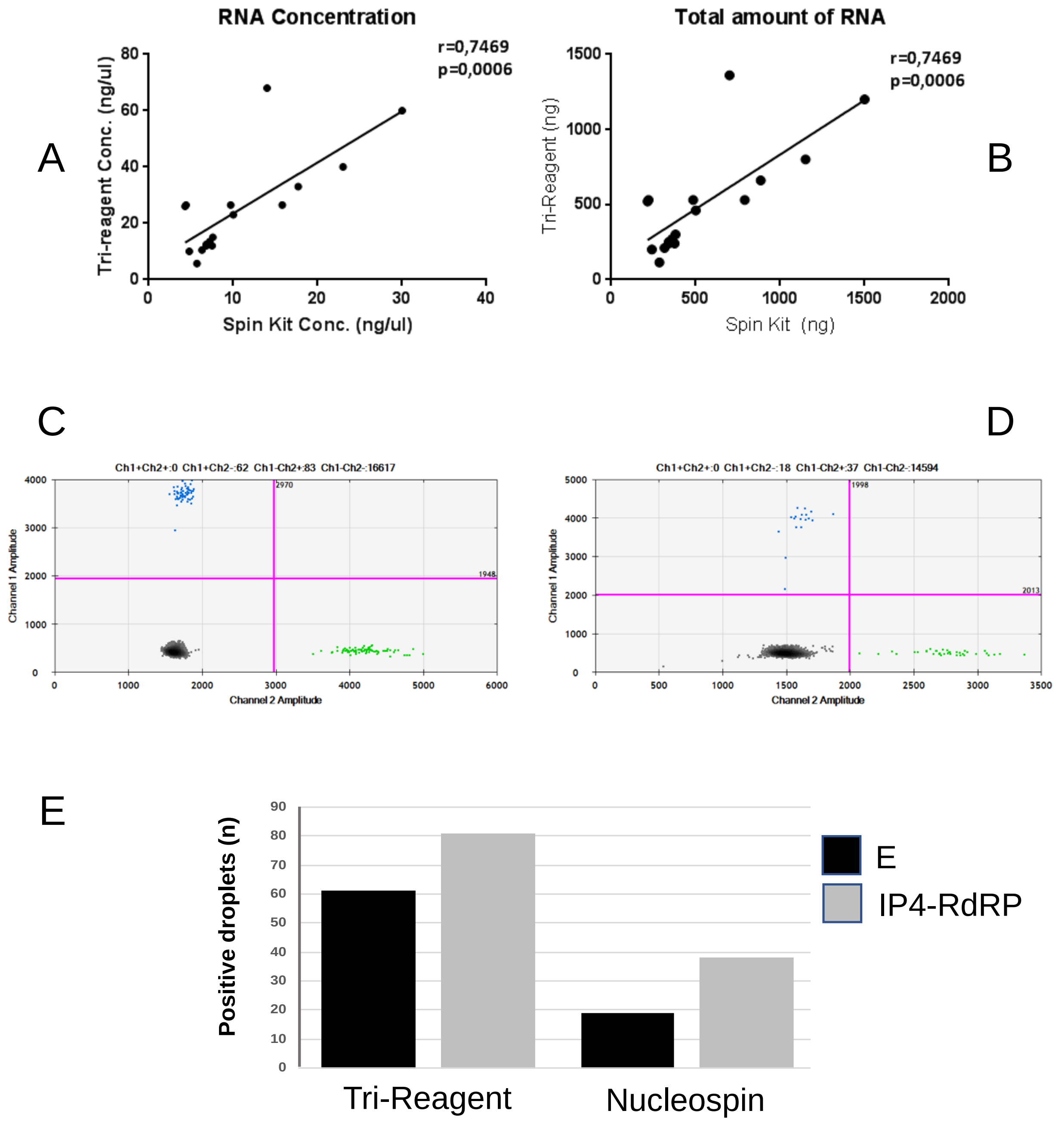
A
RNA Concentration
r=0,7469
p=0,0006
Tri-reagent Conc. (ng/ul)
Spin Kit Conc. (ng/ul)
B
Total amount of RNA
r=0,7469
p=0,0006
Tri-Reagent (ng)
Spin Kit (ng)
C
Ch1+Ch2+:0 Ch1+Ch2-:62 Ch1-Ch2+:83 Ch1-Ch2-:16617
Channel 1 Amplitude
Channel 2 Amplitude
D
Ch1+Ch2+:0 Ch1+Ch2-:18 Ch1-Ch2+:37 Ch1-Ch2-:14594
Channel 1 Amplitude
Channel 2 Amplitude
E
Positive droplets (n)
E
IP4-RdRP
Tri-Reagent
Nucleospin

Kit: iScript
Added primers: none

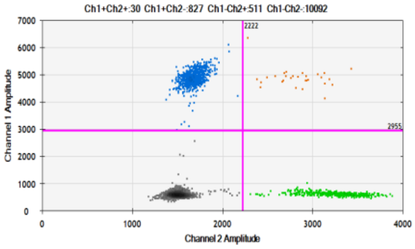

Kit: SuperScript III
Added primers: E/IP4

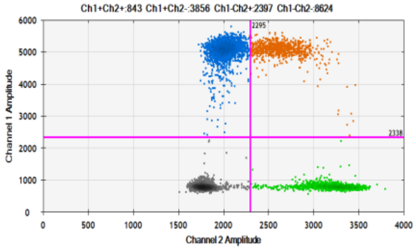

Kit: iScript
Added primers: E/IP4

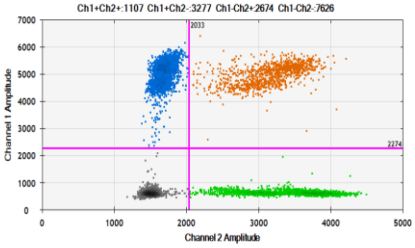

Kit: iScript Advanced
Added primers: none

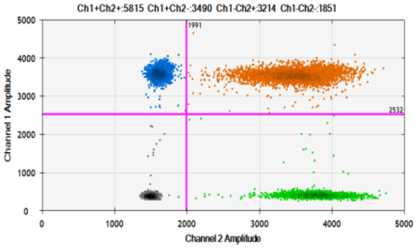

Kit: GoTaq random
Added primers: E/IP4

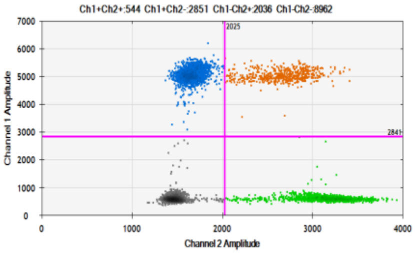

Kit: iScript Advanced
Added primers: E/IP4

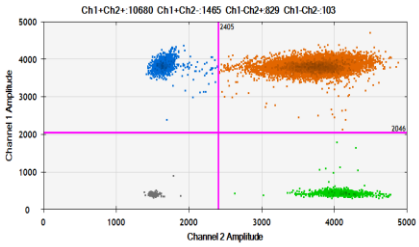

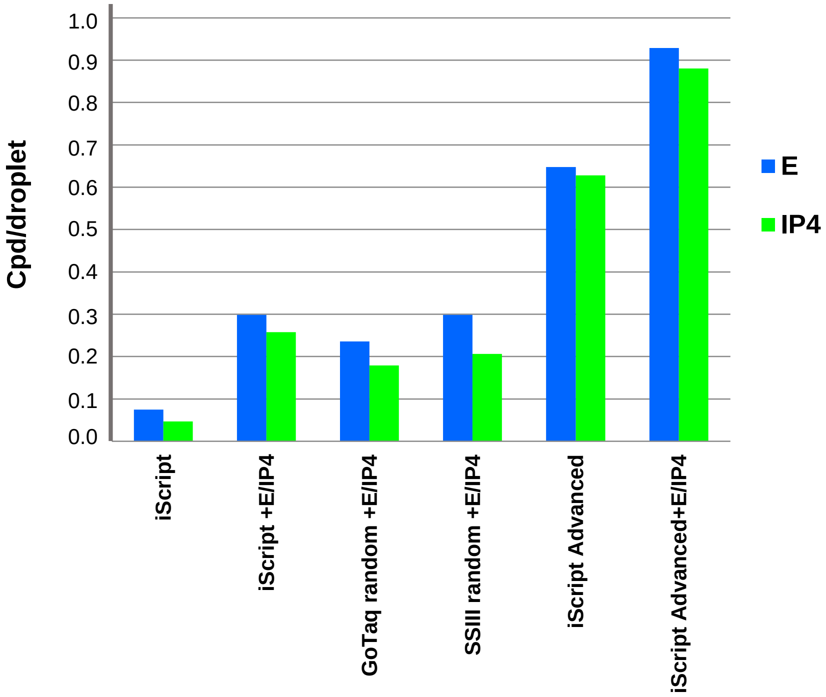

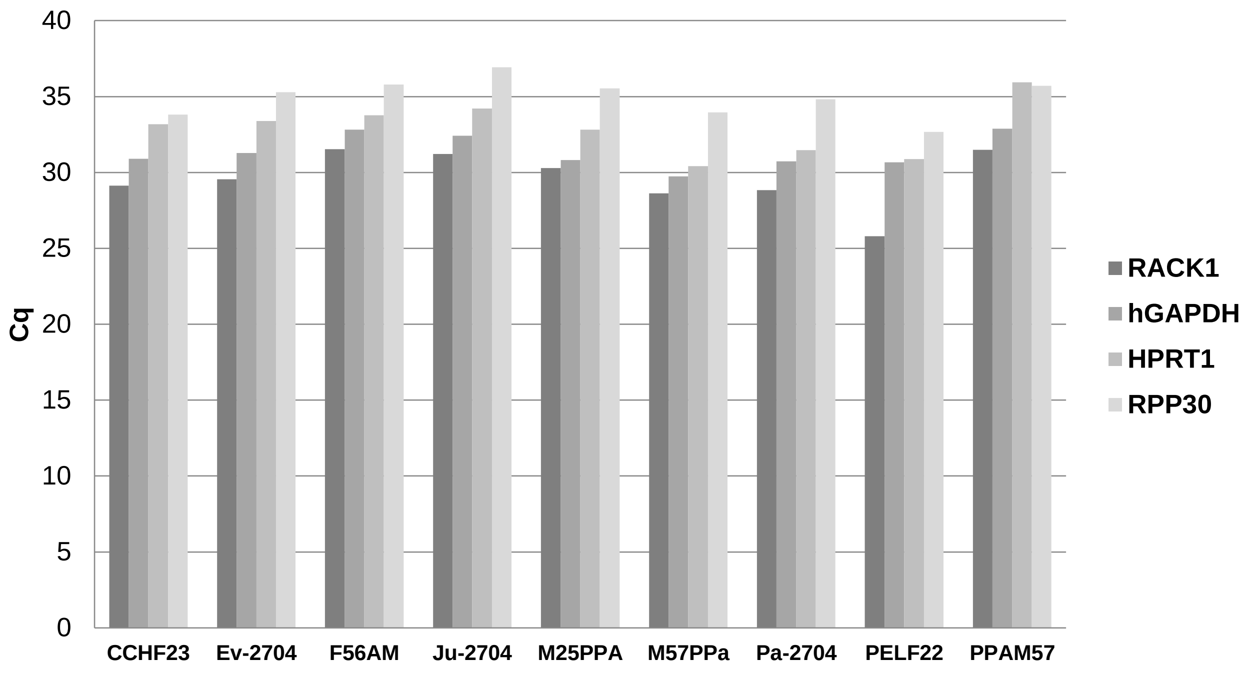
40
35
30
25
20
15
10
5
0
Cq
CCHF23
Ev-2704
F56AM
Ju-2704
M25PPA
M57PPa
Pa-2704
PELF22
PPAM57
RACK1
hGAPDH
HPRT1
RPP30

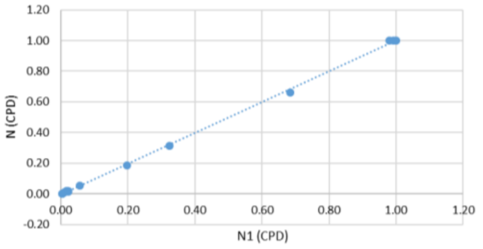
N (CPD)
N1 (CPD)
1.20
1.00
0.80
0.60
0.40
0.20
0.00
-0.20

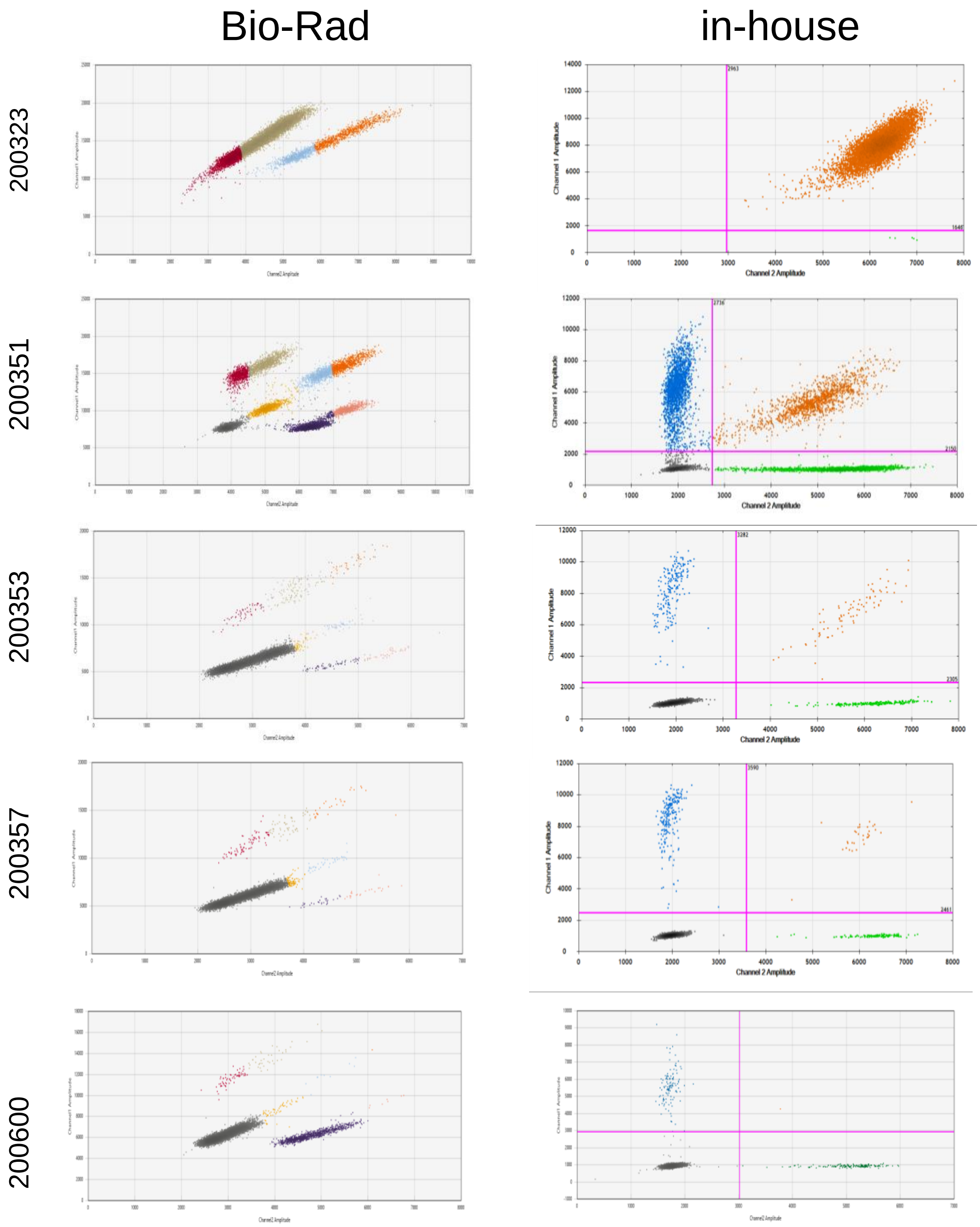
Bio-Rad
in-house
200323
200351
200353
200357
200600
Channel 1 Amplitude
Channel 2 Amplitude
