# Supplementary material for: ddPCR increases detection of SARS-CoV-2 RNA in patients with low viral loads
Source: Arch Virol. 2021 Jul 12;166(9):2529–40. doi: 10.1007/s00705-021-05149-0 (PMC8273560; doi:10.1007/s00705-021-05149-0)
Supplement: Supplementary file 2 — Supplementary file2 (DOCX 198 KB) [file 705_2021_5149_MOESM2_ESM.docx]

**ddPCR increases detection of SARS-COV-2 RNA**

**in patients with low viral loads**

*Manuscript ARVI-S-20-01274_R1*

***Supplementary information***

Agnès Marchio^1^*, Christophe Batejat^2^, Jessica Vanhomwegen^2^, Maxence Feher^2^, Quentin Grassin^2^, Maxime Chazal^3^, Olivia Raulin^4^, Anne Farges-Berth^5^, Florence Reibel^6^, Vincent Estève^5,6^, Anne Dejean^1^, Nolwenn Jouvenet^3^, Jean-Claude Manuguerra^2^, and Pascal Pineau^1*^

1: Unité « Organisation nucléaire et Oncogenèse », INSERM U993, Institut Pasteur, Paris, France

2: Cellule d’Intervention Biologique d’Urgence, Institut Pasteur, Paris, France

3: Institut Pasteur, Département de Virologie, Centre National de la Recherche Scientifique (CNRS) Unité Mixte de Recherche (UMR) 3569, Paris, France

4 : Laboratoire de Biologie Médicale, Centre Hospitalier Compiègne-Noyon, Compiègne, France

5: Laboratoire de Biologie Médicale, Groupe Hospitalier Nord-Essonne, Site de Longjumeau, Longjumeau, France

6 : Laboratoire de Biologie Médicale, Groupe Hospitalier Nord-Essonne, Site d’Orsay, Orsay, France

* corresponding authors: Mrs Agnès Marchio and Dr. Pascal Pineau, Unité « Organisation nucléaire et Oncogenèse », INSERM U993, Institut Pasteur, 28, rue du Docteur Roux, 75724 Paris, cedex 15, France, phone : 33 1 40 61 36 53, fax : 33 1 45 68 89 43, emails : [agnes.marchio@pasteur.fr](mailto:agnes.marchio@pasteur.fr), [pascal.pineau@pasteur.fr](mailto:pascal.pineau@pasteur.fr)

**Supplementary Material and Methods**

RNA extraction

In the case of Tri-Reagent LS extraction, RNA precipitation was performed in presence of 4μL Glycogen (5μg/μL, Ambion, Austin, TX, USA). Tri-Reagent-extracted RNA was resuspended in 20 μL RNAse-free water (Ambion) in presence of 1μL SUPERase-IN (20U/μL, InVitrogen, Carlsbad, CA, USA) while Nucleospin-produced RNA was resuspended in 50 μL RNAse-free water. RNA concentrations were measured with Qubit RNA HS kit (Thermo Fisher Scientific, Illkirch-Graffenstaden, France). They were ranging from 1.4 to 10 ng/mL in initial suspensions. The two extraction methods employed yielded similar outcomes concerning the quality of RNA. RNA quantities were significantly higher with the column-based method albeit at the expense of RNA concentrations a crucial aspect of SARS-CoV-2 detection (see supplementary Figure 1A-B).

2-Steps ddPCR

Reverse transcription was performed using iScript Advanced cDNA Synthesis kit for RT-qPCR with 5-14,8 μL of RNA in a final volume of 20 μL according to manufacturer’s instructions in an iCycler PCR instrument (Bio-Rad) 42°C 30min, 85°C 5min. Droplet digital PCR reactions were performed on the QX200 Droplet Digital PCR system using 5 μL of cDNA and 11μL of 2X ddPCR Supermix for probes no dUTP (Bio-Rad) in a final volume of 22 μL. PCR amplification was conducted in an iCycler PCR instrument (Bio-Rad), 10min 95°C (ramp rate of 2,5°C/sec), 40 cycles 94°C for 30s (ramp rate of 2,5°C/sec) and 59°C (ramp rate of 2°C/sec) for 1mn, 10min 98°C (ramp rate of 2,5°C/sec).

**Legends to the supplementary figures**

Supplementary figure 1: Comparison of RNA extraction methods. Concentrations (A) and quantities (B) of RNA obtained from naso-pharyngeal swabs after Tri-reagent (phenol+guanidinium thiocyanate, Y axis) or Nucleospin extraction (column, X axis). C-D; ddPCR amplification of E (Y axis) and IP4 (X axis) from SARS-CoV-2 on the same volume RNA sample. E; droplets quantification from experiments C and D

Supplementary figure 2: Representative examples of reverse transcription (RT) optimization applied to 2-steps ddPCR on E and IP4 amplimeres on SARS-CoV-2 RNA. The names of the kits tested are mentioned above each figure. All kits used random priming. Addition of specific SARS-CoV-2 primers is mentioned above the figure. 5 μL of the same RNA was used for each RT in a final volume of 20 μL. ddPCR was performed on 5 μL of RT. Primers and probe concentration are mentioned in Supplementary Table 1. Outcome of the comparison is shown on bar-chart and express in copy of target per droplet (cpd).

Supplementary figure 3: Quantification of four candidate housekeeping genes on nine naso-pharyngeal swab samples. RACK1 (receptor for activated C kinase 1) mRNA was the most expressed (lower Cq) and chosen to assess clinical sample quality.

Supplementary figure 4: (A) Correlation curve established from comparison of ddPCR experiments. Results are expressed in copy per droplet (CPD). Nucleocapsid gene detection of Bio-Rad kit (N1) was compared with the N amplimere that we used taken from T. Suo et al. (ref. #29) Similar correlation curve was drawn with N2 ass well (not shown). Pearson’s coefficients of correlation R are 0.981 and 0.967 with N1 and N2 (not shown) respectively. (B) Comparison of Bio-rad SARS-CoV-2 ddPCR kit (dEXD28563542, Cat#1200802, left panels) and in-house developed ddPCR assays on five clinical samples. Bio-Rad assay targets two different segments of N gene and RPP30 mRNA as a control while our test associates N and IP4-RdRP. The dividing lines separating droplets with different content are sometimes difficult to draw with the commercial kits.

Supplementary Table 1


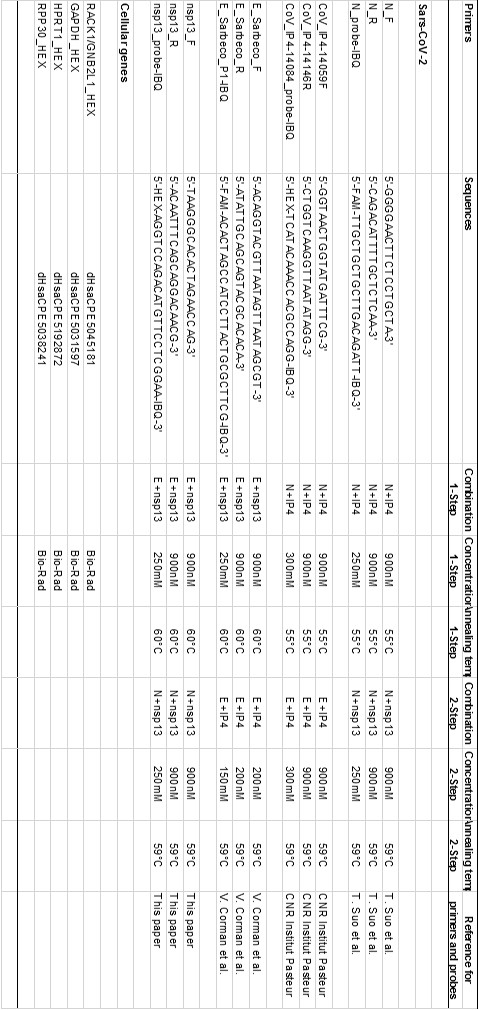


**Supplementary Table 2:** Descriptive features of the patients analyzed in the current study.
